# Improved gene tree inference from removing alignment errors both from focal genes and when training substitution models

**DOI:** 10.1093/molbev/msag182

**Published:** 2026-07-27

**Authors:** Andrew L Wheeler, Chiragdeep Chatur, Peter W Goodman, Robert C Edgar, Gavin A Huttley, Joanna Masel

**Affiliations:** Genetics Graduate Interdisciplinary Program, University of Arizona, Tucson, AZ, USA; Department of Computer Science, University of Arizona, Tucson, AZ, USA; Statistics and Data Science, University of Arizona, Tucson, AZ, USA; Independent Researcher, Corte Madera, CA, USA; Research School of Biology, The Australian National University, Canberra, Australia; Department of Ecology and Evolutionary Biology, University of Arizona, Tucson, AZ, USA

**Keywords:** phylogenetic methods, phylogenomics, detecting selection, substitution matrix, alignment confidence, homology

## Abstract

Multiple sequence alignment (MSA) is a key step in phylogenetic analysis and is prone to error. Unfortunately, algorithms that remove likely alignment errors from MSAs sometimes also remove informative residues, making phylogenetic tree inference worse. Here we present a novel MSA cleaning algorithm based on consensus between MSAs using a range of Hidden Markov Models and guide trees, named CLOAK (CLeaning On the basis of Alignment C(K)onsensus). CLOAK is a gentle filter, with a low false positive rate for removal from MSAs according to the BALiBASE benchmarks, while still removing a significant fraction of likely alignment errors. Gentle vs. stringent MSA filtering methods are appropriate for different tasks. We assess methods based on their ability to bring the gene trees of single copy orthologs closer to the accepted species tree. Amino acid substitution models trained on filtered MSAs improve gene tree inference, with stricter filtering methods providing the biggest model improvements. In contrast, it is gentler filtering of single gene MSAs that provides additional improvements to gene tree inference, with CLOAK performing best.

## Introduction

Multiple sequence alignment (MSA) is an important step in processing sequence data, prior to performing evolutionary inference. MSA attempts to identify homologous sites (Morrison et al. 2015), allowing the use of models of the evolutionary history of substitutions at each site. The alignment of non-homologous sites will propagate to many types of downstream analysis that assume homology, including inference of phylogenetic trees and of selection (Wong et al. 2008). Estimates of positive selection are particularly sensitive to the alignment method used, and alignment errors can inflate false positive rates for positive selection (Schneider et al. 2009; Fletcher and Yang 2010; Jordan and Goldman 2012). The inference of phylogenetic trees can also depend on the alignment method used (Wong et al. 2008). Tree accuracy can be improved by co-inferring the MSA with the phylogenetic tree, thus appropriately accounting for MSA uncertainty (Liu et al. 2009; Shim and Larget 2018). Unfortunately, this increases computational expense and does not scale well to larger datasets. Another option is to abandon likelihood-based approaches in favor of distance-based phylogenetics whose distance metric comes from a Hidden Markov Model (HMM) rather than a fixed MSA (Bogusz and Whelan 2017). However, the two-step approach of first generating an MSA and then taking it as fact while performing evolutionary analysis remains standard practice.

Given that MSA errors are inevitable (Landan and Graur 2009), an attractive approach is to filter out residues that are likely to be misaligned, prior to downstream analysis. Unfortunately, alignment filtering can counterproductively lead to worse performance on phylogenetic inference (Tan et al. 2015; Portik and Wiens 2021) and selection detection (Spielman et al. 2014). For a single gene tree (rather than a species tree based on a large, concatenated alignment), inadvertently removing even a small number of correctly aligned sites further reduces the availability of already scarce information.

MSAs are also used as training data for empirical substitution models, which specify the rates with which each nucleotide, amino acid, or codon evolves into each alternative state. Misaligned amino acids might introduce systematic errors into substitution models, eg inflating the inferred rate of unlikely substitutions. Substitution rates might also become inflated for amino acid pairs that are most likely to be aligned as the least bad option within a highly diverged sequence for which alignment confidence is low. Indeed, alignment uncertainty has been found to affect fit of substitution model (Spielman and Miraglia 2021). To the best of our knowledge, the impact of filtering errors out of training MSAs for substitution models has not been assessed.

Substitution models enable the use of likelihood-based methods to infer trees (Felsenstein 1981), and are also used in some distance-based methods (Bogusz and Whelan 2017). Substitution model choice thus impacts phylogenetic inference. While common practice is to use a tool such as ModelFinder (Kalyaanamoorthy et al. 2017) to choose among existing substitution models, the QMaker program within IQ-Tree recently made it straightforward to train custom substitution models (Minh et al. 2021). Here, we test whether removing alignment errors from the MSA training data input into QMaker improves subsequent gene tree inference.

The earliest approaches for MSA filtering focused on removing entire columns from an alignment. Gblocks removes poorly conserved columns that are found near gaps or are contiguous with other poorly conserved sites (Castresana 2000). TrimAl eliminates columns that fall below a score threshold, using several possible criteria such as number of gaps and residue similarity (Capella-Gutiérrez et al. 2009). Other methods score how phylogenetically informative a column is (Dress et al. 2008), randomness or entropy (Criscuolo and Gribaldo 2010; Kück et al. 2010), or alignment uncertainty (Penn et al. 2010).

Because these methods use an “all or nothing” approach for each column within an alignment, they will be too strict in a column where a subset of the sequences in a MSA are well aligned, but other sequences are poorly aligned. Deleting whole columns at such sites can result in removing information about closely related species, which is disproportionately found within rapidly evolving regions. These include intrinsically disordered regions, which tolerate a high rate of indels (de la Chaux et al. 2007; Khan et al. 2015). Tasks such as phylogenetic inference that are sensitive to loss of information are likely to benefit from a gentler approach that removes clear errors while retaining as many correctly aligned sites as possible.

Other methods identify stretches of likely non-homologous characters within each sequence. PREQUAL is a popular “pre-alignment” option that uses a HMM to identify stretches of sequence that are unlikely to be homologous to any other sequence in the dataset (Whelan et al. 2018). This approach is focused on removing frameshift errors from sequencing, assembly errors creating mosaics, and/or unique insertions, rather than on removing alignment errors.

Divvier (Ali et al. 2019) and HmmCleaner (Di Franco et al. 2019) attempt to identify incorrectly aligned sequences by using probabilistic pair HMMs to discern pairwise homology, rather than whole-column homology. These programs are influenced by earlier tools such as Zorro (Wu et al. 2012), and PSAR (Kim and Ma 2014). Divvier offers the option to retain additional phylogenetic information by “divvying,” meaning splitting some columns into two or more internally well aligned subsets; we refer to this as Divvier-d. An alternative setting in Divvier uses an approach closer to HmmCleaner, retaining only the largest subset; we refer to this as Divvier-pf. TAPER takes a different approach, searching for sliding windows of sequence that the alignment suggests are unusually divergent relative to what is expected for that species (Zhang et al. 2021). An important question is whether even these methods remove too much true phylogenetic signal, thus worsening gene tree inference.

Consensus among alternative alignments is another potentially useful source of information. Alignment consensus tools such as MergeAlign (Collingridge and Kelly 2012), and M-Coffee (Wallace et al. 2006) do not filter out any residues, but generate one consensus alignment from an ensemble.

Alternatively, MSA uncertainty can be propagated, and consensus taken later, eg among trees. This might be an effective approach to filter out random errors in an alignment, but if there are also significant systematic errors across the variant MSAs, these would require filtering. This approach will also substantially increase computational cost.

Here we propose identifying likely alignment errors based on departure from consensus within an ensemble of alternative alignments. This is implicit in Divvier's and HmmCleaner's use of HMM confidence. We also include another source of alignment uncertainty: that in the “guide tree” that specifies the order in which sequences are added during MSA construction. The guide tree is often a neighbor joining or UPGMA tree that groups together more similar sequences (Feng and Doolittle 1987). Variation in which guide tree is used can contribute substantial variability in the resulting MSA (Zhan et al. 2015). The alignment cleaning tool GUIDANCE2 generates a set of perturbed alignments by leveraging uncertainty in the guide tree. It combines this with simple penalties for alignment gaps, rather than a full HMM approach, to compute reliability scores. This enables unreliable alignments to be filtered out, with the option of filtering only parts of columns (Sela et al. 2015).

Here we develop a new consensus-based approach to alignment filtering. We look for consensus among alternative MSAs corresponding to perturbations of both the HMM and the guide tree, using an ensemble of alignments generated by Muscle5 (Edgar 2022). Pairings that appear inconsistently across these alignments indicate uncertainty and hence suggest unreliable alignment. In this light, we introduce a novel algorithm, named CLOAK, which removes pairings that fail to appear in all members of the ensemble of alignments (see Materials and Methods). For maximal information retention, we adopt the practice of divvying (Ali et al. 2019).

We assess the value of filtering, both in the construction of substitution models and in the inference of a gene tree, in terms of whether the resulting gene tree of a single copy ortholog is closer to the approximate ground truth we expect from a known mammalian species tree (with incomplete lineage sorting [ILS] responsible for some residual noise). We are therefore able to test how MSA cleaning affects the output of interest, namely the accuracy of the gene tree.

Best practices with respect to alignment filtering might depend on the application. We expect substitution model inference, which uses a large set of training MSAs, to tolerate aggressive filtering approaches that also remove a significant number of true homologies, in order to maximize the accuracy of residues that remain. For inferring gene trees, losing any well-aligned residues could harm the already limited information available for inference, requiring a lighter touch to filtering. Here we identify best practices, judged according to bringing inferred ortholog trees closer to the known species tree.

## Results

### CLOAK removes fewer correctly aligned residues than other alignment filtering tools

**Figure 1:**
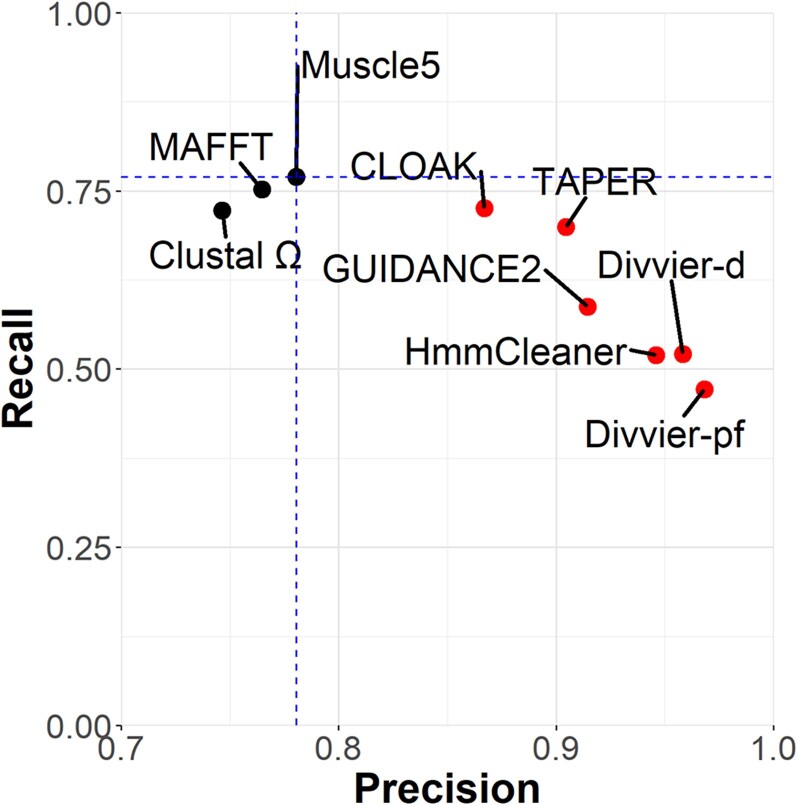
CLOAK removes a significant fraction of false pairings, with minimal loss of true information. The recall, or true positive rate, is the fraction of aligned pairs in the core region of the BAliBase reference alignment that are recovered. The precision, equal to 1 minus the false discovery rate, is the number of aligned pairs matching the BAliBase reference divided by the total number of aligned amino acid pairs in the BAliBASE core alignment. Different alignment programs are shown as black points, and different filtering methods are shown in red. Each of the filtering methods uses the Muscle5 alignment as its starting point, with distance from the blue horizontal and vertical dashed lines highlighting the extent to which true and false positives are filtered.

One way to assess an MSA is to compare it to a benchmark that putatively represents the ground truth alignment. A good MSA, or a well-filtered MSA, will increase the number of pairings that match the ground truth, while decreasing the number of pairings that do not. We use BALiBASE, whose alignments are based on protein structure, as our benchmark (Thompson et al. 2005). We evaluate alignments, both before and after filtering, using a precision-recall curve. Here precision on the *x*-axis is equal to 1 minus the false discovery rate in which amino acids are aligned in a manner not found in BALiBASE, and recall on the *y*-axis represents the rate that (presumed correct) BALiBASE pairings are recovered (Fig. 1). We confirm previous results indicating that Muscle5 outperforms MAFFT and Clustal Omega, with superiority indicated by both higher precision and higher recall (Edgar 2022). Therefore, we use Muscle5 alignments as our starting point, prior to filtering.

HmmCleaner combines lower precision with similar recall to Divvier—we therefore do not recommend HmmCleaner. Using GUIDANCE2 to remove residues with a confidence score <0.9 (see Materials and Methods) is similarly inferior to TAPER, with an only slightly higher precision at the cost of substantially worse recall. CLOAK, TAPER, Divvier-d (with divvying), and Divvier-pf (with partial filtering) all fall along a reasonable curve, where the choice of filtering program among these options may vary among applications, depending upon what balance of false positives (precision) and false negatives (recall) each application calls for.

Divvier-pf removes the most error at the cost of losing more correct homologies, making it the best option for applications that are highly sensitive to error, but which have abundant available data such that data loss is tolerable. We expect substitution model inference to fall into this category. CLOAK has the gentlest touch, removing less error but removing almost no pairings scored as correct by BALiBASE; CLOAK may be more suited for applications with highly limited available data, such as inferring single gene phylogenies.

### Alignment filtering lowers inferred rates of less mutationally accessible substitutions

**Figure 2:**
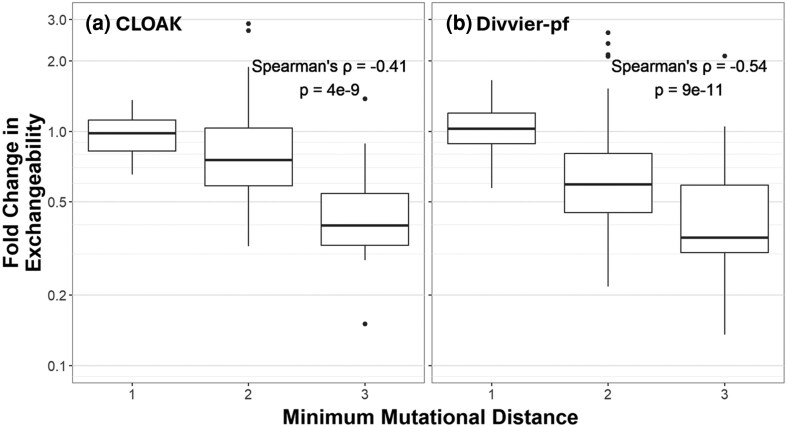
Rates of implausible substitution drop more after filtering alignments with the stricter Divvier-pf than with the gentler CLOAK. Implausible substitutions are those that require a minimum of 2 or 3 nucleotide point mutations. Exchangeabilities come from substitution models trained on a filtered vs. unfiltered alignments of mammalian orthologs (see Materials and Methods). Boxes show 25% and 75% quartiles, whiskers show 95% confidence intervals, and the central tendency line shows the median.

In the standard genetic code, some amino acid substitutions require only a single nucleotide substitution, while others require two or three point mutations (in the absence of insertions or deletions, which occur at lower rates [Chen et al. 2009]). To avoid the confounding influence of amino acid frequencies, we quantify the accessibility of substitutions corresponding to different mutational accessibilities according to “exchangeabilities.” The flux of events in which amino acid *A* evolves into amino acid *B* is equal to the frequency of *A* times the rate $k_{AB}$, and *B* evolves into *A* with flux $Bk_{BA}$. An exchangeability is defined as $r_{AB}=k_{AB}/\bar{B}=k_{BA}/\bar{A}$. This means that at equilibrium $\left\{\bar{A},\bar{B}\right\}$, the fluxes in both directions are both equal to $\;r_{AB}\bar{A}\bar{B}$. Exchangeabilities tend to be lower between amino acid pairs that are less mutationally accessible (Fig. S1). However, we expect MSA errors to introduce systematic errors that make a proportionately larger contribution to mutationally inaccessible and hence rare substitutions. Indeed, as predicted, filtering MSAs preferentially lowers the inferred exchangeabilities of less accessible substitutions (Fig. 2). Again, as predicted, this effect is weaker with a gentler filter such as CLOAK (Fig. 2a, Spearman's *ρ* = −0.41), and stronger with stricter filters such as Divvier-pf (Fig. 2b, Spearman's *ρ* = −0.54). The intermediate strength filters Taper and Divvier-d also follow the expected dose-response across the range of filtering strengths, with Spearman's *ρ* of −0.46 and −0.50, respectively.

### Filtering improves tree inference

The most important test of alignment filtering is not whether it brings alignments closer to a still-uncertain benchmark, but whether it improves inference, eg of phylogenetic trees. We use the accepted mammalian species tree (Upham et al. 2019) as approximate ground truth to assess the accuracy of gene trees of mammalian single copy orthologs, noting that even with perfect gene tree inference, there would be some residual differences due to ILS.

**Figure 3:**
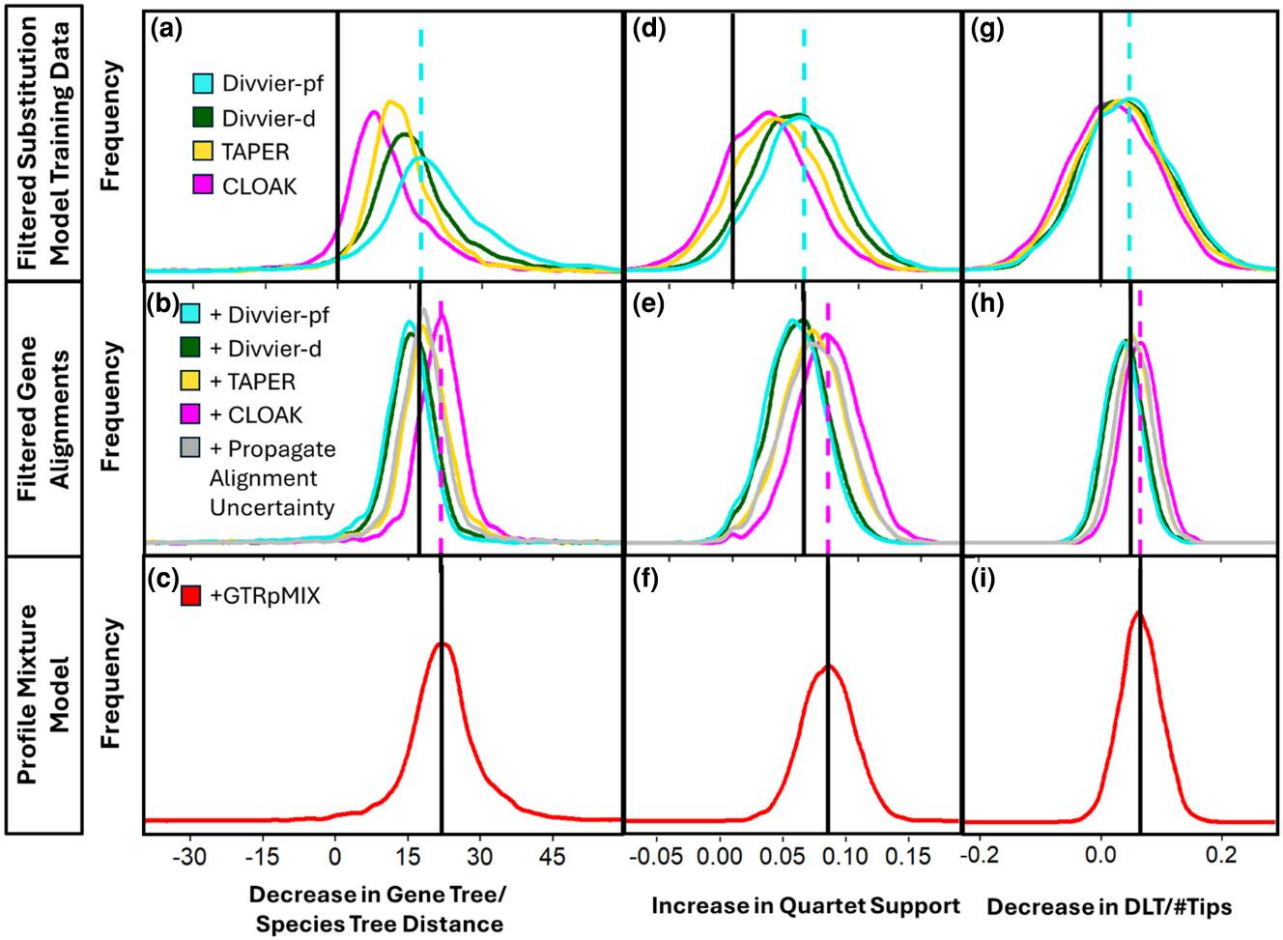
Filtering of MSAs improves tree inference. We measure the similarity of each mammalian single copy ortholog tree to the mammalian species tree. A rightward shift indicates increased similarity following filtering relative to the mean prior to filtering (vertical black line). The figure shows the distribution of pairwise differences calculated for each gene. The first column (a to c) shows decreases in the Lin–Rajan–Moret distance (Lin et al. 2012). The second column (d to f) shows the increase in the proportion of gene tree quartets that agree with the species tree topology. The third column (f, h, and i) shows decreases in DLT events inferred by reconciling each gene tree with the species tree. Baseline gene trees were inferred using unfiltered alignments for individual genes, as well as a substitution model trained on unfiltered data. All effect sizes and *P*-values can be found in Table 1; the Lin–Rajan–Moret distance produced the most statistical power. By all three metrics, the more stringent the filtering on substitution model training data, the greater the gain in gene tree accuracy (top row, a, d, and g). That is Divvier-pf produced the greatest accuracy gains, followed by Divvier-d, then TAPER, then CLOAK. Gentle filtering of single gene MSAs yields additional gene tree improvements (middle row, b, e, and h). Baseline trees in the middle row are the best case from the panel above, using unfiltered single gene alignments and a substitution model trained on Divvier-pf filtered data. Trees improved when filtering gene alignments with CLOAK and to a lesser extent TAPER, as well as when propagating alignment uncertainty to produce a consensus tree downstream. However, gene trees become worse following more stringent filtering with Divvier-d or Divvier-pf. Using the profile mixture model GTRpMIX (bottom row, c, f, and i), provides only marginal improvement in Lin–Rajan–Moret distance, with no significant change in quartet support or DLTs.

When using substitution models trained on filtered data, gene trees are more similar to the corresponding species tree (Fig. 3a, d, and g; alignments to the right of the vertical black line are improved by filtering). We get more statistical power using the Lin–Rajan–Moret distance (Lin et al. 2012; Fig. 3, first column; see *P*-values in Table 1) than from using the proportion of gene tree quartets (Estabrook et al. 1985; Day 1986) that agree with the species tree topology (Mutti et al. 2025; Fig. 3, middle column), or the number of duplication, loss, and transfer (DLT) events inferred by gene tree-species tree reconciliation (Fig. 3, right column). The Lin–Rajan–Moret distance is a weighted extension of the more commonly used Robinson–Foulds distance metric (Robinson and Foulds 1981) that offers superior discrimination and robustness to small changes, such as moving a single leaf (Lin et al. 2012).

**Table 1:** Effect sizes and significance of all comparisons in Figs. 3 and S2.

	Lin-Rajan-Moret distance		Quartet support		DLTs/Tip	
	Mean change	Paired *t*-test	Mean change (in percentage points)	Paired *t*-test	Mean change	Paired *t*-test
Filtered substitution model training data						
CLOAK	−7.9	*P* < 2e−16	2.6	*P* < 2e−16	0.021	*P* < 2e−16
Taper	−11.4	*P* < 2e−16	3.5	*P* < 2e−16	0.032	*P* < 2e−16
Divvier	−15.1	*P* < 2e−16	4.8	*P* < 2e−16	0.042	*P* < 2e−16
Divvier-pf	−17.3	*P* < 2e−16	5.7	*P* < 2e−16	0.047	*P* < 2e−16
Filtered gene alignments						
CLOAK	−3.5	*P* = 5e−14	1.9	*P* = 5e−12	0.015	*P* = 7e−6
Taper	−1.2	*P* = 3e−9	0.8	*P* = 3e−5	0.003	*P* = 0.06
Divvier	2.6	*P* = 2e−14	−0.5	*P* = 0.0009	−0.016	*P* = 5e−5
Divvier-pf	3.0	*P* < 2e−16	−0.9	*P* = 9e−5	−0.022	*P* = 9e−6
Propagate uncertainty	−1.5	*P* = 7e−10	0.9	*P* = 4e−6	0.009	*P* = 0.007
Profile mixture model						
GTRpMIX	−1.2	*P* = 8e−4	0.03	*P* = 0.08	−0.0006	*P* = 0.2
Mammal-specific model						
Compared withQ.Pfam	−1.4	*P* = 7e−9	0.06	*P* = 0.02	0.005	*P* = 0.3

The best-performing substitution model comes from applying the strictest filter, Divvier-pf, to the substitution model training data (Fig. 3, top row). Applying any of the four high-performing filtering methods (chosen above on the basis of BALiBASE) to substitution model training data leads to some improvement in gene trees.

Filtering the individual MSAs used for gene tree inference yielded additional improvement when using the gentler CLOAK and TAPER filters, but not when using the stricter Divvier-d or Divvier-pf (Fig. 3b, e, and h; vertical black line indicates the use of Divvier-pf in generating substitution models, additional improvement is indicated by a further shift to the right). The gentlest filtering option, CLOAK, performed best (pink histogram), although the additional reduction in Lin–Rajan–Moret distance is only ∼20% of the magnitude of that already obtained from using Divvier-pf filtered data to train substitution models.

Propagating the uncertainty represented by the set of 16 Muscle5 alignments into 16 trees tended to slightly improve the resulting consensus gene tree (gray histogram in Fig. 3b, e, and h), but the improvement is smaller than that obtained by using the same 16 alignments within CLOAK to filter the MSA. This suggests the existence of systematic error across all 16 MSAs, rather than merely random error.

Profile mixture models, which use a distribution of amino acid frequency vectors across sites, have been shown to perform better than the LG matrix (Banos et al. 2024). We inferred a profile mixture model from our Divvier-pf filtered training MSA dataset, then refitted the profile mixture on the CLOAK filtered test set gene alignments. This procedure yielded only marginal improvement according to Lin–Rajan–Moret distance, and no significant change according to quartet support or number of duplications, losses, and transfers, relative to a comparable filtering pipeline without use of a profile mixture (Fig. 3c, f, and i, red histogram vs. vertical black line).

**Figure 4:**
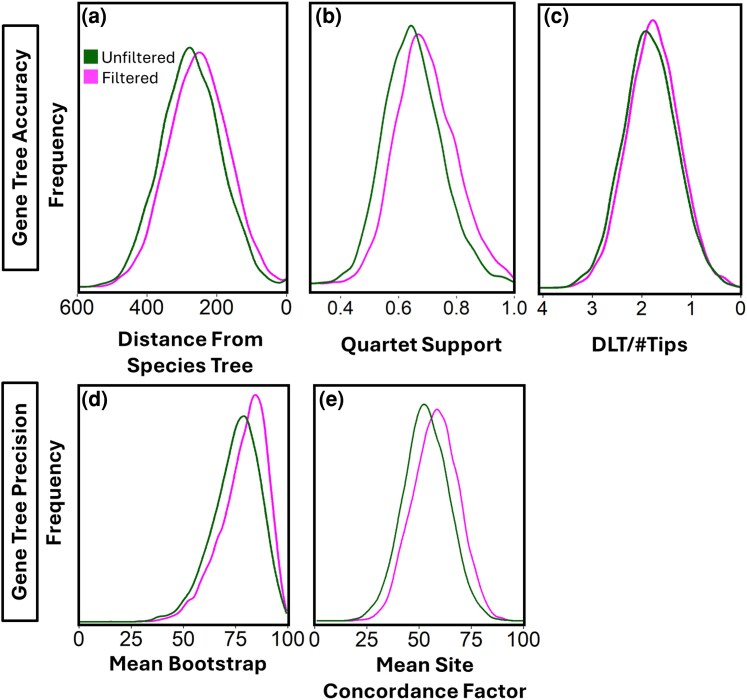
Absolute improvement in tree quality is modest relative to high variance in gene tree quality. Green distributions show the absolute gene tree quality scores prior to any filtering of either the gene alignments or the substitution model training data (the means of each distribution in Fig. 4a/b/c correspond to the vertical black lines in Fig. 3a/d/g). Pink distributions show the same scores for gene trees inferred using gene alignments filtered with CLOAK, and a substitution model trained on data filtered with Divvier-pf (corresponding to the best practice pink curves in Fig. 3b/e/h). Lin–Rajan–Moret distance is used in (a). Measures of tree confidence also show modest improvement (d: difference in means = 3.4%, e: difference in means = 4.6%) following the same filtering regime; all panels produce *P* < 2e−16 in a paired *t*-test.

Figure 4 shows absolute tree quality with no filtering, and with our best-performing filters, whereas Fig. 3 shows change in tree quality. The relatively small shifts in Fig. 4a to c illustrate that our statistical power in Fig. 3 stems from its reliance of paired analyses—variation among genes is high. This is unsurprising, eg genes vary in length and hence information content.

When ground truth in the form of a species tree is unavailable, metrics of tree confidence such as bootstrap confidence scores are sometimes used to assess tree quality. Mean bootstrap scores reflect genuine tree improvements in our analysis (Fig. 4d). Another approach to quantify precision is to use site concordance factors, quantifying the fraction of informative sites in an alignment that support the tree topology at each node (Mo et al. 2023). Bootstrap scores are inflated when there is genuine incongruence in a large dataset (Rokas and Carroll 2006); site concordance factors avoid this issue. Mean site concordance factors also reflect genuine tree improvements in our analysis (Fig. 4e).

### ILS, not alignment error, limits the accuracy of species tree inference

Our analysis is restricted to single copy orthologs, but these remain subject to ILS. ILS is when two speciation events that occur in quick succession create gene trees that are genuinely different from species trees. For example, around 30% of human genes are more or equally related to their gorilla ortholog as to their chimpanzee ortholog (Mailund et al. 2014; Rivas-González et al. 2023). Poor gene tree estimation will both score ILS where it does not occur and fail to score it where it does. Since most genes do not have ILS, this inflates the amount of detected ILS overall. Cleaning dropped the apparent frequency of ILS from 2,145 (32%) to 2,124 (31%) out of 6,769 trees. While this effect is in the expected direction, it is not statistically significant (Pearson's *χ*^2^ test, *P* = 0.8).

**Figure 5:**
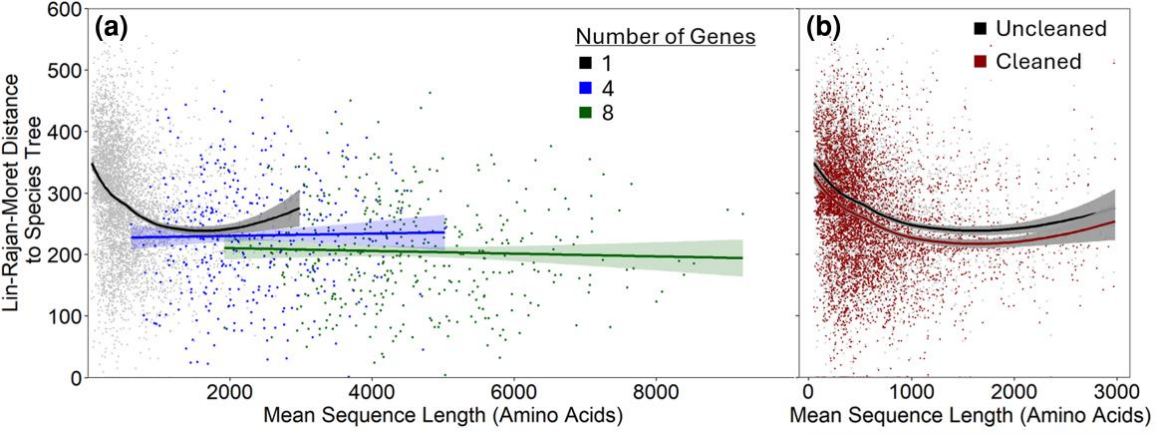
Accurate species tree inference depends more on how many genes are concatenated than how many sites or whether alignments are filtered. *X* axes show the average length of the uncleaned sequences within each alignment. In (a), four or eight genes were each sampled 400 times without replacement from the set of 5,769 mammalian ortholog alignments (gray points). Uncleaned alignments were used. The black curve is a loess regression for the larger dataset. We used linear regression for the smaller four and eight gene sets, none of which yielded statistically significant slopes. Shaded areas show 95% confidence intervals. Mean distances to the species tree were significantly different between groups (*P* < 2e−16 for one-gene vs. four-gene, *P* = 3e−9 for four-gene vs. eight-gene, *t*-test with unequal variance). b) Visualizes the same Lin–Rajan–Moret distances shown in Fig. 4a, this time in the context of sequence length as a driver of variation among genes. Each gray point corresponds to a tree from an unfiltered alignment, and each blue point to a tree from a filtered alignments.

One approach to species tree inference is to use only genes for which ILS is considered less likely, eg by avoiding immune system genes for which balancing selection is more likely, as well as genes in regions of low recombination (Rivas-González et al. 2023). However, there is a trade-off with sequence quantity, and no approach is guaranteed to avoid ILS altogether. One extreme strategy within this trade-off is to concatenate many single copy orthologs, on the basis that ILS will average out. Figure 5a shows how the advantage of concatenating single copy orthologs, without selecting them eg on the basis of high local recombination rates, exceeds that from merely having a single longer gene, presumably because of averaging out ILS. The benefit from alignment cleaning is in turn even smaller than the benefit from using a long rather than a short single gene, once gene length exceeds a modest threshold (Fig. 5b). Figure 5 thus suggests that ILS poses a far larger challenge to species tree inference than alignment quality does. For this reason, we do not expect the alignment filtering approaches used in this paper to improve species tree inference using concatenated alignments in taxonomic groups comparable to mammals. Alignment cleaning matters when information is extremely limited and must be used optimally, as is the case when constructing a gene tree.

### Model fit does not predict model performance

A program such as ModelFinder is normally used to choose the best fitting substitution model under the presumption that this will generate the best tree (Kalyaanamoorthy et al. 2017). However, if we do that with our CLOAK filtered gene alignments, the best-performing *Q* matrix (Divvier-pf filtered) is chosen less often (for 2,405 out of 6,769 genes or 36%) than the *Q* matrix trained on unfiltered data. This corresponds to the fact that CLOAK is a much gentler filter than Divvier-pf, and corresponding alignments are thus closer to unfiltered alignments.

### Pre-computed *Q*

It is striking that ModelFinder chooses a *Q* matrix that yields inferior trees, suggesting that ModelFinder should not be used to choose *Q*. Assessing the importance of eg taxon-specific *Q*, is generally based on goodness of fit (Minh et al. 2021; Dang et al. 2022; Dang and Vinh 2024), which is a soft endpoint, rather than our hard endpoint of gene tree accuracy.

The most thorough alternative, following our pipeline here, is to train a custom *Q* matrix using QMaker (Minh et al. 2021) on data filtered with Divvier-pf. The reason for repeating this work would be that *Q* matrices differ among taxa (Minh et al. 2021; Dang and Vinh 2024), and on the basis of protein structure (Koshi and Goldstein 1995; Le and Gascuel 2010). However, these differences are less important than cleaning training data. For general use, we trained a *Q* matrix based on cleaned data from the Pfam dataset. Combining the exchangeabilities of this QC.Pfam matrix trained on cleaned Pfam data with raw (uncleaned) mammalian amino acid frequencies performed almost as well as the QC.mammal matrix trained on cleaned mammalian data (Fig. S2). GTRpMIX produced similarly negligible improvements in Fig. 3c, f, and i. Partitioning on the basis of AlphaFold-predicted structure (Goodman et al. in prep.) did not even statistically improve gene trees.

If taxonomic and structural differences in *Q* are important only to model fit and not to downstream tree quality, then an easy alternative to using ModelFinder is to use a pre-computed *Q*, trained on filtered data, that is a rough taxonomic match, and to replace its equilibrium amino acid frequencies with those in the dataset of interest. To assist with this, we provide a variety of pre-trained “QC” matrices via IQ-Tree 3; these include options trained on the Pfam dataset, on bacteria, and on archaea, as well as the mammalian *Q* that has been our primary object of study above.

## Discussion

The best approach to filtering MSAs depends on the application. We find that strictly filtering training data can significantly improve substitution models in a way that leads to more accurate phylogenetic tree inference. While previous studies have found that filtering the scarce MSA data used in gene tree inference is actually harmful (Tan et al. 2015; Portik and Wiens 2021), we find that this is only true of strict filters, and that gentler filtering improves gene tree inference. We also present a new MSA filtering algorithm, CLOAK, based on consensus between alignments that perturb both HMMs and guide trees, that performs well as a gentle filter. We make new recommendations for best practice. We recommend against using ModelFinder to select *Q* and instead recommend either the a priori use of a “QC” matrix previously trained on cleaned data, or for thoroughness, the use of QMaker on training data tailored to the application of interest.

These recommendations from our work are downstream and distinct from early pipeline steps to remove sequences that may not be homologous at the whole-sequence level, or that are of poor sequence quality. A pre-alignment tool such as PREQUAL (Whelan et al. 2018) can be helpful in such cases.

One concern when filtering alignments, especially when using the divvying function of CLOAK or Divvier-d, is that adding more gaps might make downstream inference worse than it would be if the column were simply omitted. Gaps are treated in most likelihood models as real amino acids or nucleotides whose identity is unknown, represented by an equilibrium frequency vector of possible amino acids (Felsenstein 1981). Gappier sequences will therefore tend to be inferred to be too similar to each other, and to sequences with typical amino acid usage. However, despite the fact that the divvying procedure of CLOAK introduces more gaps than many alternative approaches, it outperforms other methods for tree inference.

Our recommendations imply a claim about which masked alignment is “better” than another. What it means for an alignment to be better is not trivial (Morrison et al. 2015). A common interpretation of an MSA is that characters in the same column are homologous, meaning they are descended from a shared ancestral nucleotide or amino acid. The alignment, including “-” characters, can represent point mutations, insertions, or deletions at that site.

However, for some evolutionary events, homology cannot be represented in MSA format. Following a tandem duplication, two characters in one sequence are homologous to one character in another. In an MSA, this would be represented as an insertion, with only one of the duplicates correctly shown as homologous to the original. Similarly, inversions can only be represented in an MSA as either a string of consecutive substitutions, or as a deletion and an insertion. In neither case does the MSA correctly identify the homology of the characters within the inversion. In amino acid or codon alignments, insertions and deletions at the nucleotide level might not match codon boundaries. For example, an out-of-frame deletion of 3 nucleotides creates a new codon formed from a hybrid of two other codons, and therefore an amino acid that is homologous to two ancestral amino acids (García Mesa et al. 2024). Likelihood approaches will interpret the amino acid MSA as a point mutation plus an in-frame deletion.

With none of the plausible alignments are able to capture the true evolutionary history at every site, we pragmatically judge alignment filtering methods based on their downstream ability to recover a more accurate gene tree. In this framework, the goal of filtering MSAs is not necessarily to make alignments “more homologous,” but to remove sites that impede accurate downstream inference.

Counterintuitively, the best fitting substitution model according to likelihood metrics is not the same as the model that best improves gene tree inference. GTRpMIX (Banos et al. 2024), related structural partition models (Goodman et al. in prep.), and taxon-specific *Q* matrices (Minh et al. 2021; Dang et al. 2022; Dang and Vinh 2024) all improve likelihood, but fail to substantially improve gene trees (Figs. 3c, f, i and S2, and Goodman et al. in prep.). In contrast, filtering *Q*'s training data makes fit-likelihoods worse, but improves downstream trees.

We suspect that these improvements come from lengthening the time to model saturation. Saturation occurs when so many substitutions occur at the same site that their number cannot be determined, and is typically underestimated (Philippe et al. 2011). Alignment errors are enriched for mutationally inaccessible and hence implausible substitutions, inflating their exchangeabilities. On what should be a long branch, inflated rates of mutationally inaccessible substitutions can act as evolutionary shortcuts, explaining a change via a single rare substitution rather than via a more realistic multi-step process, thereby shortening the long branch. Better resolution of long branch lengths, ie increasing the amount of evolutionary time before saturation kicks in, helps us better resolve ancient speciation or duplication events.

Gently filtering gene alignments to remove spurious implausible substitutions can also improve gene trees. Accurate models of rate heterogeneity are critical to accurate phylogenetic inference (Yang 1996; Soubrier et al. 2012). Alignment errors will mimic fast evolving sites, inflating the weight and rate parameters for fast rate categories. Removing the worst offending sites should reduce the impact of alignment errors on the rate model and produce a rate model that more accurately captures the true distribution of evolutionary rates across sites.

The optimal approach to filtering MSAs may be different for downstream applications other than gene tree inference. For species tree reconstruction, data is generally not limiting enough for filtering to be critical, especially in the face of other challenges such as ILS. However, there should be no harm in using cleaned QC matrices rather than uncleaned *Q* matrices, and indeed better resolution of gene trees should help more accurately quantify ILS. Gene trees have many other applications, especially in prokaryotes, with some of the most extreme applications being to the Last Universal Common Ancestor (Moody et al. 2024; Wehbi et al. 2024).

If the benefit from using cleaned QC comes from lengthening time to saturation, then we expect more impact on deep time events, potentially including the problem of long branch attraction even in a species tree. Indeed, by assessing on mammalian trees, we chose a challenging test set in which the advantages provided by our method were less likely to be impactful.

Tests for positive selection are very sensitive to alignment errors, which tend to produce false positives (Schneider et al. 2009). A strict filtering method such as Divvier-pf might therefore improve selection detection. Indeed, HmmCleaner was able to reduce the rate of false positives in a simulated dataset (Di Franco et al. 2019). However, other simulation based studies have shown mixed results for using GUIDANCE to improve selection detection, with some showing improved accuracy (Privman et al. 2012) but others finding little benefit (Jordan and Goldman 2012; Spielman et al. 2014).

While we built CLOAK with amino acid alignments in mind, CLOAK can accept any fasta formatted alignment files, including nucleotide alignments, or even structural alignments generated by FoldMason using the 3Di alphabet (Gilchrist et al. 2024). A similar consensus-based approach should be applicable to other alignment types, such as codon alignments, but CLOAK currently only accommodates fasta format. All that is needed is a set of alternative MSAs that capture uncertainty from an appropriate range of sources.

Most of the improvement we saw to gene tree accuracy came from using superior substitution models, rather than from filtering the gene MSA. This suggests that substitution model improvements are a promising avenue of research for improving phylogenetic inference. In the best case, a custom substitution model experimentally determined via deep mutational scanning of a specific protein dramatically outperform traditionally inferred models (Bloom 2014). Even without custom experimental data, a well-chosen alignment filter such as Divvier-pf can contribute to ensuring high quality data during the training of higher-performing substitution models.

## Materials and methods

### CLeaning on the basis of alignment C(K)onsensus

**Figure 6:**
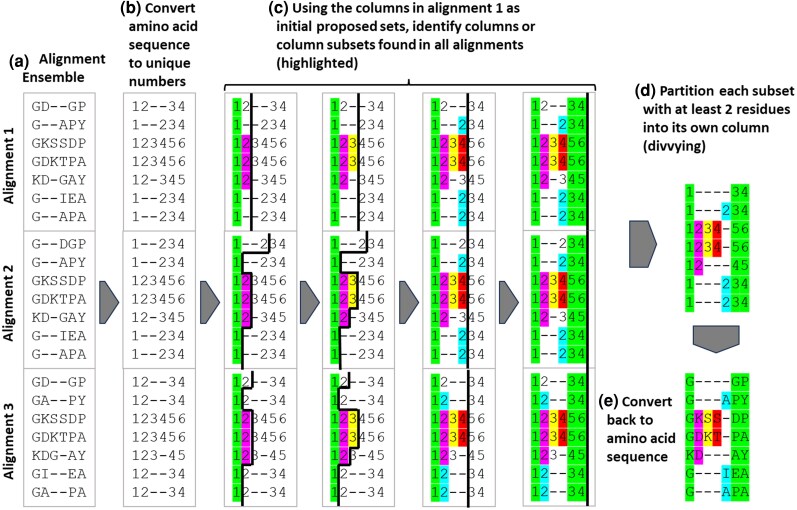
A schematic representation of our algorithm. Muscle5 produces 16 alternative alignments of the same amino acid sequences; we show only three for simplicity (a to c). To avoid confusion among amino acids of the same type, we first number each amino acid according to its position in the protein sequence (b). Our aim is to identify amino acid groupings that appear together in every alignment, albeit potentially in different columns in different alignments. We progress through each column of the first alignment, proposing that all its amino acids be grouped together. We then progress through each other alignment until we find the same amino acids, and check whether they also appear together in every other alignment (c). When all alignments agree, but regarding some but not all amino acids in a column (colored sets), we divvy (Ali et al. 2019) into multiple columns to represent consistent amino acid groupings (d). We discard subgroupings of only a single amino acid because these contain no information. Finally, we convert numbers back into amino acids, to return a filtered, divvied alignment (e).

CLOAK inputs a set of variant alignments of the same sequences and searches for consensus among them. We generate the input alignments for CLOAK using Muscle5, whose “-stratified” option conducts three perturbations of its core HMM and three perturbations of its core guide tree, the combination of which produces 16 alternative alignments. Our algorithm retains each pair of amino acids in the same column if and only if they are found together (in the same column) in all 16 alternative alignments. If multiple subsets of amino acids within a column are found together across all alignments, but the whole column is not, the subsets are split into multiple columns that are each retained (divvying). Columns with a single character (indicating a character ie not consistently grouped with any other character across all 16 alternative alignments), are removed from the alignment. Our method is shown in Fig. 6.

### Benchmarking alignments with BALIBase

We re-aligned the sequences in the BALiBASE version 3.0 dataset (Thompson et al. 2005), including the N- and C-termini, using Muscle version 5.1 (Edgar 2022), MAFFT version 7.471, and Clustal Ω version 1.2.4. We filtered each Muscle5 alignment using CLOAK, Divvier (using the -divvygap option, and with or without the partial filtering option) (Ali et al. 2019), HmmCleaner version 0.243280 (Di Franco et al. 2019), TAPER (Zhang et al. 2021), and GUIDANCE version 2.01 (Sela et al. 2015). GUIDANCE2 uses a set of variant alignments (by perturbing the guide tree), from which it calculates confidence scores for each residue. We used variant alignments from Muscle5, and masked all amino acids in the alignment with residue scores <0.9. For all programs, default parameters were used except where specified.

We compared each filtered or unfiltered alignment to the structurally aligned region of BALiBASE (presumed to be ground truth), excluding the N- and C-terminal annotated regions, to quantify precision and recall (Fig. 1). Our script for this was adapted from that used in Ali et al. (2019) to assess Divvier (Simon Whelan, script provided via personal correspondence), which compares all pairwise homologies within a region shared by two alignments.

### Mammalian orthologs

To minimize the degree to which the true gene tree differs from the species tree (due to paralogy, ILS, and horizontal gene transfer), we use a curated set of mammalian orthologs. We used a set of 6,769 orthologous mammalian protein sequences across 190 species from the OrthoMaM database version 12 (Allio et al. 2024), using only genes with at least one sequence in each of the seven mammalian taxonomic groups annotated in the OrthoMaM database (euarchontes, glires, laurasiatheria, afrotheria, marsupialia, monotremata, and xenarthra). Starting with the unfiltered alignments from OrthoMaM, we re-aligned the sequences using Muscle5 (Edgar 2022). We used a subsample of 1,000 of these loci to train amino acid substitution models, and the remaining 5,769 loci as a testing set to infer single gene phylogenetic trees.

### Amino acid substitution models

Amino acid substitution models describe a continuous time Markov process via a rate matrix **Q**, containing the rates at which each of the 20 amino acids are substituted for each other. In a time-reversible model, the fluxes between each member of an amino acid pair are equal in both directions. **Q** can then be conveniently expressed, using fewer parameters, as the product of a symmetric matrix of exchangeability rates ($r_{ij}=r_{ji}$) and a vector of equilibrium amino acid frequencies (π), where $q_{ij}=r_{ij}\pi_{j}$ such that $q_{ij}\pi_{i}=q_{\;ji}\pi_{j}$. QMaker takes equilibrium amino acid frequencies π to be equal to current amino acid frequencies (Minh et al. 2021). The diagonal entries of **Q** ($q_{i,i}$), are set such that each row sums to 0.

After inferring a substitution matrix with QMaker, we manually perform the normalization of **Q** that is normally done within IQ-Tree. We divide the matrix by a factor


$$\mu =-\overset{20}{\underset{i=1}{\sum }}\;\pi_{i}q_{i,i},$$

making the total number of substitutions per unit time equal to 1. We calculated normalized exchangeability values (for Figs. 2 and S1) from normalized **Q** matrices as:


$$r_{i,j}=r_{\;j,i}=q_{ij}/\pi_{j}$$

Because alignment filtering removes some amino acids more than others (eg preferentially removing amino acids that tend to be rapidly evolving), substitution models trained on filtered data will have equilibrium amino acid frequencies that do not match those of the original alignments. To avoid this problem, we generate **Q** by multiplying the exchangeability matrix trained on filtered MSAs by the equilibrium amino acid frequency vector calculated from the testing dataset. In some but not all cases, this testing dataset was also filtered, generally with a milder filter.

### Taxon specific substitution models trained on filtered data

We trained additional substitution models on filtered data from three other datasets. First, we used the Q.pfam training dataset from Minh et al. (2021) containing 6,654 protein alignments from version 31 of the Pfam database (El-Gebali et al. 2018). For our archaeal substitution model, we downloaded sequences from the High-quality Automated and Manual Annotation of Proteins (HAMAP) database of 2,388 protein families (Lima et al. 2009). For bacteria, we used the training dataset of Dang et al. (2024), which they took from HAMAP. The bacterial dataset contains a sample of 1,000 protein families with all non-bacterial and duplicate sequences removed. For archaea, we similarly used all 688 protein families containing archaeal sequences, and removed any non-archaeal or duplicate sequences. Our 688 archaeal protein alignments included 117,305 sequences and 565,369 sites. For all three datasets, we re-aligned all protein families using muscle5 (Edgar 2022), and then filtered them using Divvier-pf (Ali et al. 2019). We then inferred substitution models for each dataset using QMaker (Minh et al. 2021).

### Comparison of gene trees to species trees

Our mammalian species tree is a subset of the vertlife maximum clade credibility tree (Upham et al. 2019), restricted to the 190 species in the OrthoMaM ortholog dataset (Allio et al. 2024). The vertlife tree was constructed from a supermatrix of 31 gene alignments, drawing data from 4,098 mammalian species.

We inferred gene trees from Muscle5 gene alignments of the testing subset of our mammalian ortholog sequences. Tree inference was performed using IQTree2 (Minh et al. 2020), manually specifying which substitution matrix *Q* to use. For each gene, we selected a model for rate heterogeneity among sites using the ModelFinder program in IQTree2 (Kalyaanamoorthy et al. 2017), which was usually a free rate model. To propagate alignment error, we inferred 16 alternative gene trees from an ensemble of 16 alternative Muscle5 alignments, and used the M-Coffee package of the T-Coffee distribution to infer a single consensus tree (Notredame et al. 2000).

We calculated the Lin–Rajan–Moret distance (Lin et al. 2012) between gene trees and the reference species tree using the *tree_distance* function in the cogent3 python library (Huttley et al. 2025). The proportion of gene tree quartets that match the species tree was calculated with ASTRAL v.5.7.1 (Zhang et al. 2018) using:

java -jar astral.5.7.1.jar -i {gene treefile} -q {species treefile}

The number of DLT events in each gene tree was inferred with a parsimony approach implemented in Notung v.2.9.1 (Stolzer et al. 2012), and normalized by the number of tips.

### Gene tree precision

We inferred bootstrap scores based on 1,000 replicates per gene tree, using the ultrafast bootstrap application in IQTree2 (Hoang et al. 2018). Site concordance factors are the proportion of informative sites in an alignment that support the tree topology at each node. We calculated site concordance factors for each gene tree using IQTree2 (Mo et al. 2023), and took the mean across all branches in the tree.

## Supplementary Material

msag182_Supplementary_Data

## Data Availability

CLOAK is available both as an option within Muscle5 and as a stand-alone python script. Muscle5 can be found at https://www.drive5.com/muscle/, and CLOAK can be run within Muscle5 using the -cloak option. The stand-alone version of the CLOAK program, instructions for installing and running CLOAK alone and within Muscle5, and all scripts used for data analysis, can be found at https://github.com/phylowheeler/CLOAK.
