# Supplementary material for: Improved gene tree inference from removing alignment errors both from focal genes and when training substitution models
Source: Mol Biol Evol. 2026 Jul 27;43(8):msag182. doi: 10.1093/molbev/msag182 (PMC13449614; doi:10.1093/molbev/msag182)
Supplement: Supplementary file 1 [file msag182_supplementary_data.pdf]

Supplemental Figures for

“Improved gene tree inference from removing alignment errors both from focal genes and when training substitution models”

Andrew L. Wheeler, Chiragdeep Chatur, Peter W Goodman, Robert C Edgar, Gavin A Huttley, Joanna Masel

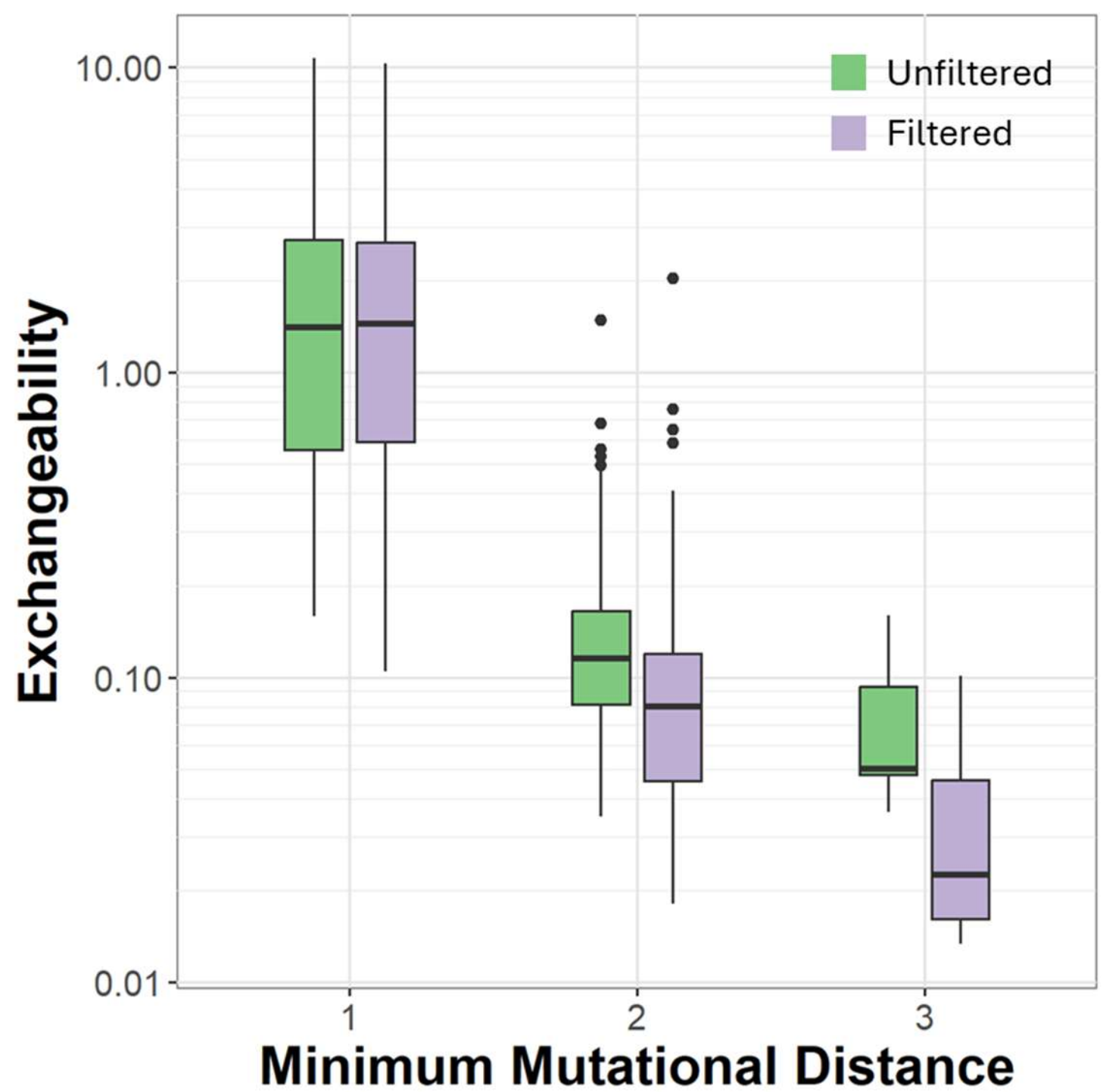

Supplementary Figure 1: Exchangeabilities tend to be lower between amino acid pairs that are less mutationally accessible. The effects of filtering with Divvier-pf (green vs. purple) are smaller but still appreciable relative to exchangeability differences on the basis of minimum mutational distance. Minimum mutational distance indicates the minimum number of nucleotide point mutations required to achieve a given amino acid substitution. Exchangeabilities come from substitution models trained on a filtered vs. unfiltered alignments of mammalian orthologs (see Methods). Boxes show 25% and 75% quartiles, whiskers show 95% confidence intervals, and the central tendency line shows the mean.

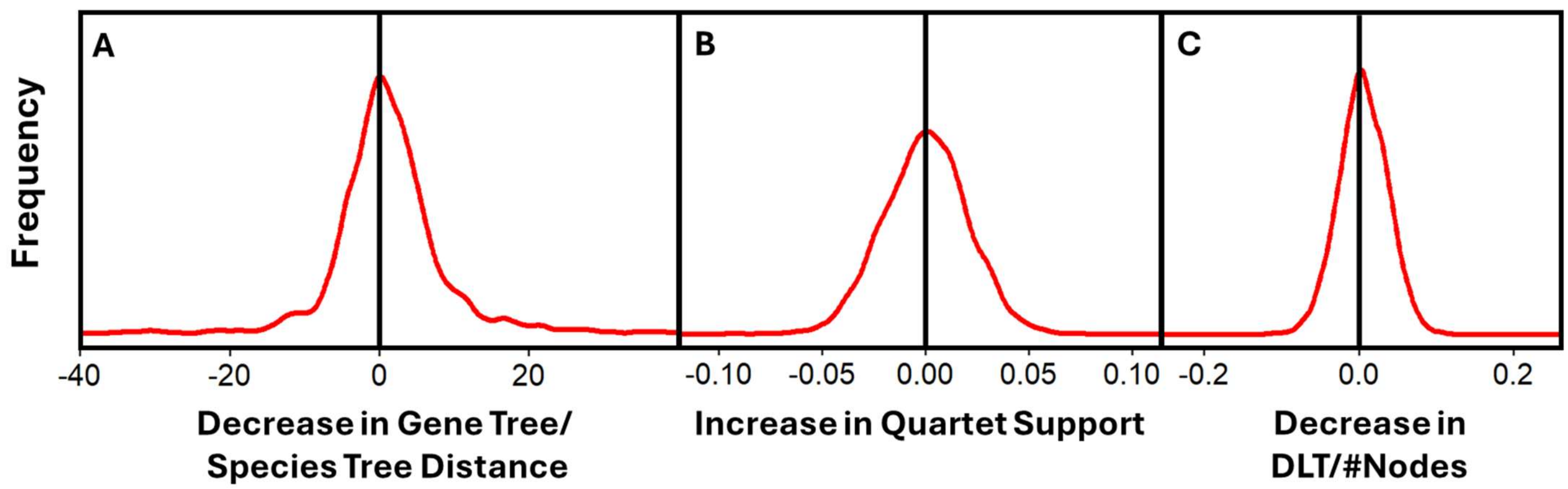

Supplementary Figure 2: A mammal specific substitution model provides only small improvements to gene tree inference. The figure shows the degree to which mammalian ortholog trees move closer to the mammalian species tree when using QC.mammal rather than QC.pfam for gene tree inference. Distributions of pairwise differences in each metric are shown across the set of gene. Mean values greater than 0 indicate better performance of QC.mammal. Mean values and corresponding paired t-test values (7e-9, 0.02, and 0.3 for panels A-C, respectively) are shown in Table 1.
